# Longitudinal Changes of Axial Length and Associated Factors in Congenital Ectopia Lentis Patients

**DOI:** 10.1155/2022/4032283

**Published:** 2022-06-07

**Authors:** Jingxin He, Zhangkai Lian, Qianzhong Cao, Zhenzhen Liu, Charlotte Aimee Young, Xinyu Zhang, Danying Zheng, Guangming Jin

**Affiliations:** ^1^State Key Laboratory of Ophthalmology, Zhongshan Ophthalmic Center, Sun Yat-Sen University, Guangdong Provincial Key Laboratory of Ophthalmology and Visual Science, Guangdong Provincial Clinical Research Center for Ocular Diseases, Guangzhou 510060, China; ^2^Department of Ophthalmology, Third Affiliated Hospital, Nanchang University, Nanchang, Jiangxi Province, China

## Abstract

**Purpose:**

To investigate the longitudinal changes and associated factors of axial length (AL) in congenital ectopia lentis (CEL) patients.

**Methods:**

In this retrospective study, medical records of CEL patients were reviewed from January 2014 to December 2019 at the Zhongshan Ophthalmic (ZOC) in China. Patients were divided into the surgery group and the nonsurgery group. Data of refractive power, best-corrected visual acuity (BCVA), and intraocular pressure (IOP) as well as ocular biometrics including AL, corneal curvature, white-to-white (WTW), and central corneal thickness (CCT) were collected at baseline and each follow-up visit. Multiple linear regression was performed to assess the potential associated factors for axial length growth in congenital ectopia lentis patients.

**Results:**

Compared with the nonsurgery group, the change rate of AL among children aged 3 to 6 years old was slower in the surgery group (0.443 ± 0.340 mm/year vs. 0.278 ± 0.227 mm/year, *P* < 0.05). However, no statistically significant difference for the change rate of AL was detected between the surgery group and the nonsurgery group (*P* > 0.05) among patients aged 7 years or older. For the surgery group, the results of the linear regression model showed that a higher change rate of AL was associated with younger age (older age: *β* = −0.009, 95% CI: −0.014 to −0.003, and *P*=0.002) and worse baseline BCVA (logMAR) (*β* = 0.256, 95% CI: 0.072 to 0.439, and *P*=0.007). As for the nonsurgery group, younger baseline age (older age: *β* = −0.027, 95% CI: −0.048 to −0.007, and *P*=0.01) and longer baseline AL (*β* = 0.073, 95% CI: 0.023 to 0.122, and *P*=0.006) were associated with a higher change rate of AL.

**Conclusions:**

The AL change rate was clearly associated with age both in the surgery group and in the nonsurgery group. Intervention strategies such as surgery should be performed earlier for CEL that meets the surgical criteria. Worse baseline BCVA and longer baseline AL are associated factors that would affect the growth rate of AL in the surgery and nonsurgery group, respectively.

## 1. Introduction

Congenital ectopia lentis (CEL) is defined as the dislocation of the lens from its natural position [1] which usually occurs bilaterally and is often associated with inherited connective tissue disorder such as Marfan syndrome, Weill–Marchesani syndrome, homocystinuria, and Ehlers–Danlos syndrome [2]. Dislocation of the lens could cause high refractive error such as irregular astigmatism, high myopia, or high hyperopia, which usually leads to not only visual impairment but could also lead to diplopia and strabismus, especially during the critical period in ocular development [3].

The methods of CEL treatment could be generally classified into conservative treatment and operative treatment. It is generally considered that patients with mild EL and transparent crystalline lenses without serious complications can be conservatively treated, that is, observation with regular follow-up and wearing spectacles or contact lenses for refractive correction. Only when a dislocated lens seriously impairs vision and quality of life, should surgical intervention be adopted [4].

Previous studies reported that both visual deprivation and optical defocus can alter the ocular growth pattern due to the rapid elongation during the ocular growth period of childhood [5, 6]. Axial length (AL), which is generally considered to be one of the primary determinants of the refractive status, could be affected by different treatment methods in children with cataracts [7]. Our previous research has shown that the eyes with CEL had a longer AL compared with the normal eyes, and this difference was more significant in children younger than 12 years old [8]. However, the influence of different therapy methods and potential associated factors on the axial growth in CEL patients has not been reported.

In the study, we aimed to analyze and compare the longitudinal changes of AL in different treatment strategies and evaluate the potential associated factors that will affect the longitudinal changes of AL for CEL patients.

## 2. Methods

### 2.1. Subjects

In this retrospective study, CEL patients were recruited from January 2014 to December 2019 from the Zhongshan Ophthalmic Center. The inclusion criteria were as follows: (1) patients with detailed clinical data and biological parameters and (2) patients were followed up for 26 months on an average (range 1–3 years) and the interval between two follow-ups was more than 3 months. The exclusion criteria were as follows: (1) patients with incomplete data; (2) preexisting ocular diseases that may influence ocular development, such as congenital glaucoma, congenital cataracts, or other ocular diseases that can lead to defocus or deprivation; (3) patients with lens dislocation due to ocular trauma, tumor, or surgery. Surgery was considered if one or more of the following criteria [9, 10] was observed: (a) best-corrected visual acuity (BCVA) was less than 0.3; (b) complicated with severe cataract; (c) monocular diplopia; (d) progressive subluxation of the lens affecting the pupillary axis with or without elevation of intraocular pressure (IOP); and (e) with serious complications, such as secondary glaucoma, corneal endothelial decompensation, and/or retinal detachment.

Patients were divided into the 2 groups according to the baseline age: 3 to 6 years old (3–6 y) and 7 or more years old (≥7 y). In each age group, patients were further divided into the surgery group and the nonsurgery group based on their treatments. For the operative treatment group, the eye that underwent surgery was included for the study, and for the conservative treatment group, only the right eye was included for the study. For the nonsurgery group, the baseline age and baseline axial length were defined as the age of initial diagnosis of CEL. For the surgery group, age at the time of the surgery and the preoperative axial length were regarded as the baseline age and baseline axial length of statistical analysis, respectively. Subgroup analyses were carried out in accordance with the baseline age and baseline SE. Basic characteristics such as age, gender, medical history, and hospitalization time were extracted. Parameter and refractive data of ocular including anterior chamber depth (ACD), astigmatism, intraocular pressure (IOP), white-to-white corneal diameter (WTW), central corneal thickness (CCT), best-corrected visual acuity (BCVA), and spherical equivalent (SE) were also recorded.

The study followed the tenets of the Declaration of Helsinki and was approved by the Institutional Review Board of the Zhongshan Ophthalmic Center in Sun Yat-sen University (IRB-ZOC-SYSU), Guangzhou, China.

### 2.2. Surgical Technique

For patients that underwent surgery, the surgical criteria were consistent with previous studies [11, 12]. Surgery was performed by the same surgeon (Dr. DY Zheng), and patients underwent the same procedure of lens extraction and transscleral IOL fixation. In detail, two triangular scleral flaps were made at 4 and 10 o'clock posterior of the corneal limbus. A 3.0 mm clear corneal tunnel incision was made at 12 o'clock, and a continuous circular capsulorhexis (CCC) was performed. Lens extraction was performed with the capsule being held by the iris retractor, and the capsular bag was taken out after the phacoaspiration. Intraocular lens (IOL) transscleral fixation was then performed with the two IOL haptic sutured by using an 8-0 prolene suture at 2 mm posterior to the corneal limbus under the sclera flap. A 10-0 nylon suture was used to close the scleral flaps and the main corneal incision. Anterior vitrectomy was performed only in the eyes with severe vitreous prolapse.

### 2.3. Statistical Analysis

All the included data were extracted from medical records and checked by two independent investigators (JXH and ZKL). The data that follow the Gaussian distribution were analyzed by using Student's *t*-test, and the data that do not the follow Gaussian distribution were performed using the rank-sum test for between-group comparisons. *P* values less than 0.05 were considered statistically significant.

Univariate and multivariate linear regression analyses were conducted to assess potential correlation between baseline biometry variables and the AL change rate. All data analyses were performed using Stata 14.0 software (Stata Corp., College Station, TX, USA).

## 3. Results

In total, 148 eyes of 148 CEL patients were included in this study. Among them, 101 (68.2%) underwent surgery and 47 (31.8%) received conservative therapy. The demographic and clinical features of patients are presented in Table 1.

**Figure 1:**
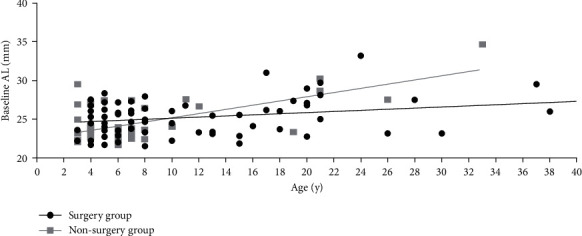
Distribution of axial length with age in the surgery group and the nonsurgery group of CEL patients.

**Figure 2:**
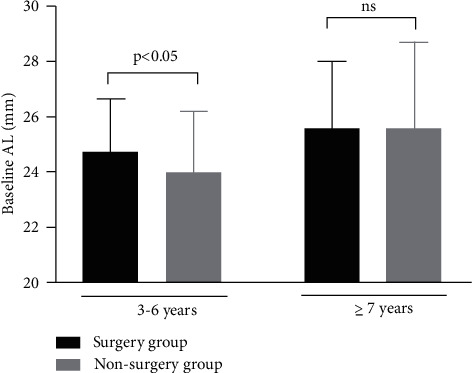
Comparison of the baseline axial length in the surgery group and the nonsurgery group of CEL patients.

**Figure 3:**
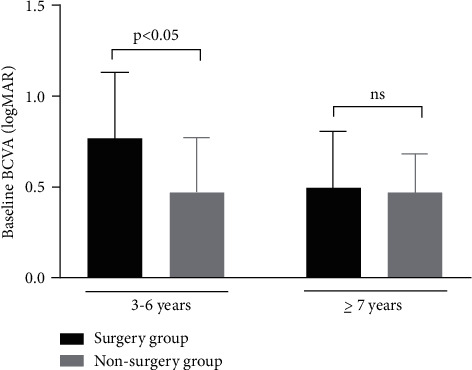
Comparison of baseline best-corrected visual acuity in the surgery group and the nonsurgery group of CEL patients.

**Figure 4:**
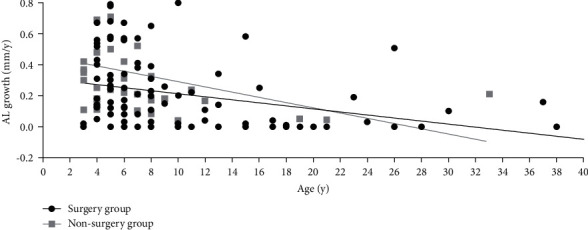
Scatter plot between the growth rate of axial length and age in the surgery group and the nonsurgery group of CEL patients.

**Figure 5:**
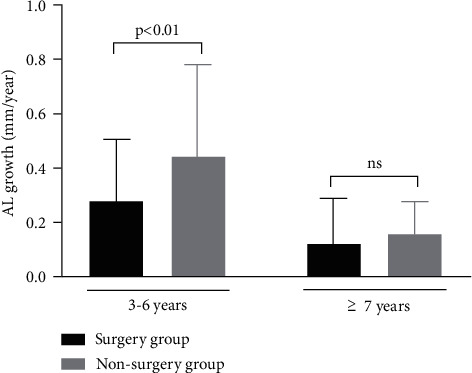
Comparison of the growth rate of axial length in the surgery group and the nonsurgery group of CEL patients.

In this study, significant differences of baseline AL between the surgery group and the nonsurgery group were found in patients aged 3 to 6 years (24.73 ± 1.90 mm vs. 24.0 ± 2.19 mm, and *P* < 0.05). In patients aged 7 years or older, no difference of baseline AL was detected between the two groups (25.6 ± 2.45 mm for the surgery group vs. 25.6 ± 3.12 mm for the nonsurgery group and *P*=0.486) (Table 2 and Figures 1 and 2). And there was a significantly worse baseline logMAR BCVA in the surgery group than in the non-surgery group (0.774 ± 0.362 vs. 0.471 ± 0.302, *P* < 0.05) for the 3 to 6 year age group, wwhile in the older age group (7 years or older), no significant difference of the baseline BCVA differences was detected between the surgery group and the nonsurgery group (Table 2 and Figure 3). For patients aged 3 to 6 years old, the increase rate of AL was 0.278 ± 0.227 mm per year after surgery in the surgery group, which was lower than that in the nonsurgery group (0.278 ± 0.227 mm/y vs. 0.443 ± 0.340 mm/y and *P* < 0.05). However, no statistically significant differences of the AL growth rate were found between the surgery subgroup and the nonsurgery subgroup in the7 years or older group (Table 2 and Figures 4 and 5). No statistically significant difference was detected for corneal astigmatism, WTW, CCT, IOP, and SE between the surgery subgroup and the nonsurgery subgroup at the baseline in the two age groups (Table 2).

Multivariate linear regression showed that the AL growth rate was associated with older surgery age (*β* = −0.009, 95% CI: −0.014 to −0.003, and *P*=0.002) and lower logMAR BCVA (*β* = 0.256, 95% CI: 0.072 to 0.439, and *P*=0.007) in the surgery subgroup. For the nonsurgery subgroup, the AL change rate was associated with older age (*β* = −0.027, 95% CI: −0.048 to −0.007, and *P*=0.01) and shorter AL (*β* = 0.073, 95% CI: 0.023 to 0.122, *P*=0.006) (Table 3). Linear regression analysis also revealed that the AL change rate was significantly associated with surgical treatment after adjusted for age and sex in all patients aged 3 to 6 years old (*β* = −0.16, 95% CI: −0.30 to −0.02, *P*=0.022).

## 4. Discussion

In the current study, we found that the average AL change rate in the surgery group was 0.28 ± 0.23 mm/year, which was slower than that in the nonsurgery group (0.44 ± 0.34 mm/year) for patients aged 3 to 6 years old. In the surgery group, age at surgery and baseline BCVA was significantly associated with axial elongation after IOL implantation for CEL patients. As for the nonsurgery group, the factors associated with axial length growth were the baseline age and baseline AL. For all patients aged 3 to 6 years old, the AL change rate was significantly associated with surgical treatment after adjusted for age and sex.

Few studies have investigated the influence of different treatment methods on axial elongation. In this study, our results show that the AL change rate in the surgery group was slower than that in the nonsurgery group for patients aged 3 to 6 years old. It was believed that surgical treatment had an obvious effect on curbing the AL elongation during the early period of eyeball development. One explanation is that surgical treatment can provide patients with a better visual quality, while conservative therapy cannot maintain a stable visual quality. It is well known that a stable visual quality is beneficial for normal eyeball development while depriving the eye of form vision will result in excessive axial elongation and myopia [5, 13–15]. Previous studies have drawn similar conclusions that in patients with CEL, the conservative treatment method such as wearing spectacles or contact lenses could lead to a high incidence of amblyopia [16], which often accompanies axial elongation.

Interestingly, no statistically significant differences were detected between the surgery group and the nonsurgery group for AL growth for the 7 years or older group. Our results were consistent with a previous study that reported that AL increased rapidly at younger age and then slowed and stabilized [17]. One possible reason is that the influence of lens dislocation on the development of AL may be stronger when the eye is undergoing the most rapid phase of axial growth in early years of life [17]. As the patient gets older, the development of axial length slows and stabilizes and the influence of lens dislocation decreases.

To explore the associated factors of AL changing in CEL patients who undergo surgery, several potential associated factors were also analyzed in this study and age and baseline BCVA were identified as factors that affect the AL elongation. For healthy children, a number of different factors including the age [17], gender [18], and BMI [19] have been identified as factors having the potential to affect the rate of AL growth. Previous studies also showed that the longitudinal growth of the AL can be divided into 3 growth periods: a rapid postnatal phase with an increase in length of 3.7–3.8 mm in the first year and a half, followed by a slower infantile phase from the 2nd to the 5th year of life with an increase in length of 1.1–1.2 mm, and finally by a slow juvenile phase lasting until the age of 13 years, with an increase of 1.3–1.4 mm [17]. For patients who had undergone surgery, our results indicated that participants with older age demonstrated a smaller degree of axial length elongation after adjusting for age and gender. This may help further verify the conclusions of previous studies that younger children were shown to undergo faster rates of axial elongation [20–22]. In addition, we found that a better postoperative BCVA was negatively associated with the AL change rate after IOL implantation. The association between BCVA (log MAR) and AL has not been widely studied. However, animal experiments showed that the visual deprivation could result in an elongation of AL and a myopic shift in the refractive state [23]. And previous studies also showed that alterations of the visual input in early life could affect axial growth of the ocular in experimental animals, and neural factors evoked by abnormal visual experience are thought to influence the growth of the posterior segment of the ocular [24].

For the nonsurgery group, multivariate analyses showed that the factors associated with the AL change rate were younger age and a longer baseline AL. It has been well demonstrated that the growth rate of the eyeball is most rapid in the first 3 to 4 years of life; then, the subsequent annual increase in length appears to be slight [17]. In Japanese youth (7 to 21 years old) with myopia, the AL elongation rate decreased with age, especially in the group older than 15 years [25]. Our results was similar to a previous study [26] conducted by Li et al. in Shanghai, which showed that AL elongation was associated with a longer AL at baseline. Thus, the baseline AL may provide a predictive factor in the axial length change in CEL patients.

Our result indicated that for patients aged 3 to 6 years, the baseline AL in the surgery group were longer than that in the nonsurgery group. In addition, the surgery group had worse BCVA than the nonsurgery group. However, for patients 7 years or older, both the baseline AL and BCVA were comparable between the surgery group and the nonsurgery group. The differences of the baseline AL and BCVA in patients aged 3–6 years between the surgery group and the nonsurgery group were probably due to the surgical criteria. As a previous study [27] reported, CEL can be managed with conservative treatments such as spectacle or contact lens correction when symptoms are mild. However, for those with severe complications, surgery would be a better treatment strategy for patients to achieve a better visual acuity. As previously discussed, significant changes in AL usually occur in CEL patients during the early period of eyeball development.

The limitations of this study are as follows: Firstly, bias may exist, for not all the patients could be reviewed consistently on a consistent follow-up schedule. Secondly, although a number of factors have been taken into consideration, some potential associated factors for AL changing were not included for analysis, which may affect the accuracy of the conclusion to some extent. Despite these limitations, this longitudinal study with a large sample size for a rare disease offers a view of evaluating and comparing an AL change between different treatment strategies and these findings could provide useful information for the treatment and management of CEL.

## 5. Conclusions

In conclusion, our results suggested that surgical treatment had an obvious effect on curbing the AL elongation during the early period (3–6 years old) of eyeball development, but little influence was observed when patients were older than 7 years of age. For younger patients who met the surgical criteria, surgery should be performed earlier to promote a normal development of AL. For CEL patients, regular follow-up should be emphasized especially for those with a longer AL, younger age, and worse BCVA.

## Figures and Tables

**Table 1:** The demographic characteristics of the included CEL patients.

	Total	Surgery group	Nonsurgery group	*P*
Median age (IQR)	6 (5–11)	7 (5–13)	6 (4–8)	0.035
Age group, *n* (%)				
3–6 years	75	48	27	0.002
≥7 years	73	53	20	
Total	148	101	47	
Male, *n* (%)	96 (64.9%)	67 (66.3%)	29 (61.7%)	0.292

**Table 2:** Baseline and the changing trend of ocular parameters in the surgery group and the nonsurgery group of CEL patients.

	3–6 years			≥7 years		
	Surgery group	Nonsurgery group	*P*	Surgery group	Nonsurgery group	*P*
Baseline						
AL (mm)	24.73 ± 1.90	24.0 ± 2.19	0.047	25.6 ± 2.45	25.6 ± 3.12	0.486
SE (D)	−5.27 ± 10.0	−7.37 ± 8.13	0.18	−3.94 ± 11.08	−11.0 ± 8.06	0.007^*∗*^
BCVA (logMAR)	0.774 ± 0.362	0.471 ± 0.302	0.001^*∗*^	0.498 ± 0.311	0.472 ± 0.212	0.367
IOP (mmHg)	14.04 ± 3.32	12.9 ± 2.58	0.075	13.7 ± 2.83	14.9 ± 3.35	0.09
Km	39.44 ± 6.43	41.1 ± 1.67	0.09	39.7 ± 8.78	41.1 ± 1.73	0.243
WTW (mm)	12.17 ± 0.57	12.14 ± 0.603	0.422	12.06 ± 0.486	12.1 ± 0.484	0.431
CCT	541.9 ± 46.0	544.4 ± 44.4	0.392	543.41 ± 45.1	545.4 ± 51.7	0.245
Change of AL (mm/year)	0.278 ± 0.227	0.443 ± 0.340	0.007^*∗*^	0.121 ± 0.168	0.156 ± 0.123	0.201

AL = axial length; *D* = diopter; SE = spherical equivalent; BCVA = best-corrected visual acuity; IOP = intraocular pressure, *y*; Km = (*K*1 + *K*2)/2; WTW = white-to-white corneal diameter; CCT = central corneal thickness.

**Table 3:** Potential associated factors for axial length growth in CEL patients.

	Surgery group				Nonsurgery group			
	Univariate regression		Multiple regression		Univariate regression		Multiple regression	
	*β* (95% CI)	*P*	*β* (95% CI)	*P*	*β* (95% CI)	*P*	*β* (95% CI)	*P*
Female	−0.046 (−0.136, 0.044)	0.310	—	—	0.065 (−0.118, 0.248)	0.475	—	—
Age (years)	−0.008 (−0.013, −0.003)	0.001^*∗*^	−0.009 (−0.014, −0.003)	0.002^*∗*^	−0.016 (−0.031, −0.002)	0.029^*∗*^	−0.027 (−0.048, −0.007)	0.01^*∗*^
AL (mm)	0.007 (−0.011, 0.025)	0.441	—	—	0.078 (0.037, 0.118)	<0.001^*∗*^	0.073 (0.023, 0.122)	0.006^*∗*^
IOP (mmHg)	0.000 (−0.012, 0.013)	0.944	—	—	−0.041 (−0.080, −0.003)	0.036^*∗*^	−0.031 (−0.064, 0.001)	0.060
BCVA (logMAR)	0.181 (0.012, 0.352)	0.036^*∗*^	0.256 (0.072, 0.439)	0.007^*∗*^	0.366 (0.114, 0.618)	0.006^*∗*^	—	—
CCT (mm)	0.001 (−0.001, 0.001)	0.426	0.001 (−0.001, 0.002)	0.080	—	—	—	—
SE (*D*)	−0.007 (−0.018,0.003)	0.174	—	—	−0.009 (−0.021, 0.002)	0.117	—	—
Km (*D*)	−0.003 (−0.025, 0.019)	0.801	—	—	−0.014 (−0.071, 0.043)	0.622	0.038 (−0.018, 0.094)	0.175

Surgery group: AL, IOP, BCVA (logMAR), CCT, SE, and Km were detected at baseline; nonsurgery group: AL, IOP, BCVA (logMAR), CCT, SE, and Km were performed at 3 months after operation.

## Data Availability

Data are available upon reasonable request.
